# Membranes Are Decisive for Maximum Freezing Efficiency of Bacterial Ice Nucleators

**DOI:** 10.1021/acs.jpclett.1c03118

**Published:** 2021-11-01

**Authors:** R. Schwidetzky, P. Sudera, A. T. Backes, U. Pöschl, M. Bonn, J. Fröhlich-Nowoisky, K. Meister

**Affiliations:** † Max Planck Institute for Polymer Research, 55128 Mainz, Germany; ‡ Max Planck Institute for Chemistry, 55128 Mainz, Germany; § University of Alaska Southeast, Juneau, Alaska 99801, United States

## Abstract

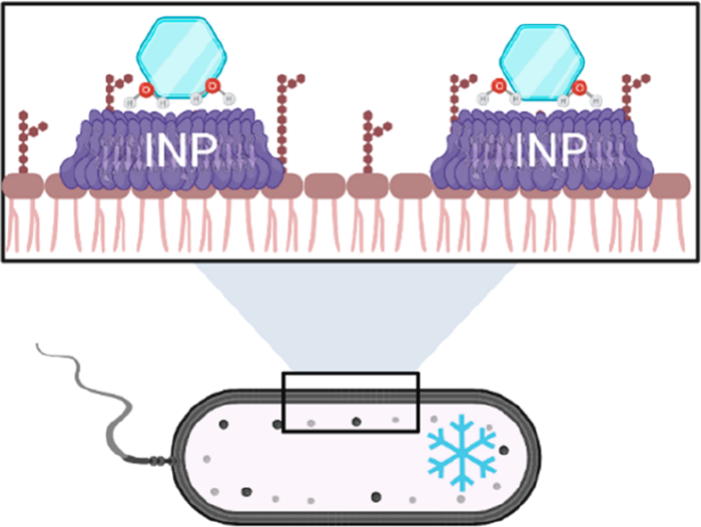

Ice-nucleating proteins (INPs) from *Pseudomonas syringae* are among the most active ice nucleators known, enabling ice formation at temperatures close to the melting point of water. The working mechanisms of INPs remain elusive, but their ice nucleation activity has been proposed to depend on the ability to form large INP aggregates. Here, we provide experimental evidence that INPs alone are not sufficient to achieve maximum freezing efficiency and that intact membranes are critical. Ice nucleation measurements of phospholipids and lipopolysaccharides show that these membrane components are not part of the active nucleation site but rather enable INP assembly. Substantially improved ice nucleation by INP assemblies is observed for deuterated water, indicating stabilization of assemblies by the stronger hydrogen bonds of D_2_O. Together, these results show that the degree of order/disorder and the assembly size are critically important in determining the extent to which bacterial INPs can facilitate ice nucleation.

The formation of ice is thermodynamically favored at temperatures below 0 °C, but the crystallization is kinetically hindered. As a result, pure water can be supercooled to temperatures as low as −38 °C, below which homogeneous ice nucleation occurs.^1^ Nature provides extraordinary examples of how to induce ice formation at much warmer temperatures. Certain ice nucleation active microbes enable ice formation at temperatures close to 0 °C, better than other organic or inorganic material.^2,3^ Ice nucleation active bacteria cause frost damage to plants, and in the atmosphere, they may glaciate clouds and influence precipitation patterns.^4,5^ The best characterized biological ice nucleators (INs) are from the plant-associated bacteria *Pseudomonas syringae*.^2,6^ The ability of *P. syringae* to facilitate ice nucleation is attributed to specialized ice-nucleating proteins (INPs) anchored to the outer bacterial cell membrane.^7,8^ The functional structure of the INPs has been reported to contain a hydrophobic N-terminal domain, a hydrophilic C-terminal domain, and a large central repeat domain presumably acting as ice nucleation sites.^9^ The proposed nucleation sites consist of arrays of STQT and ESSLT motifs, where threonine and serine are most conserved.^10^ Apart from the structural properties of the active site, the exceptional activity of bacterial INs has been reported to critically depend on the ability of the INPs to assemble into large clusters.^10−12^ Based on their activity in droplet freezing experiments, the bacterial INs are oftentimes grouped into classes A–C.^13^ Class A INs consist of large aggregates (>30 INPs) and are responsible for freezing between ∼−2 and ∼−4 °C, whereas C INs consist of smaller aggregates that induce freezing at ∼−7.5 °C.^10,13^ Class B INs are less common and responsible for freezing between ∼-5 and ∼−7 °C. The INPs are localized in the outer bacterial membrane, as demonstrated by fractionating experiments and the isolation of ice-nucleating vesicles shed from the membrane.^8,14^ The leaflet of the outer membrane of Gram-negative bacteria like *P. syringae* consists of phospholipids and complex lipopolysaccharides (Figure 1). Turner et al. suggested that phosphatidylinositol (PI) is important for ice nucleation activity as a part of the ice nucleation site and to serve as an anchor for INPs.^15,16^ In contrast, Schmid et al. reported that INPs cannot be anchored to the membrane via PI. Govindarajan et al. further showed that delipidating membranes abolished the activity of class C INs and that the addition of lipids reconstituted activity.^7^ A number of studies also revealed that chemicals that disrupt the fluidity of the membrane reduced ice nucleation activity.^2,7,17^ Recent studies further showed that environmental factors (pH, salts, antifreeze proteins) can have very different effects on class A and class C INs.^11,18−20^ Here, we use the high-throughput twin-plate ice nucleation assay (TINA) to investigate the ice nucleation activity of the lipids 1,2-dimyristoyl-3-trimethylammonium −propane (DPTAP), 1,2-dipalmitoyl-*sn*-glycero-3-phosphoglycerol (sodium counterion) (DPPG), 1,2-dipalmitoyl-*sn*-glycero-3-phosphorylethanolamine (chloride counterion) (DPPE), phosphatidylinositol (PI), lipopolysaccharides, and the effects of deuterated water, heat, and delipidation on the ice nucleation activity of *P. syringae*.^21^

**Figure 1:**
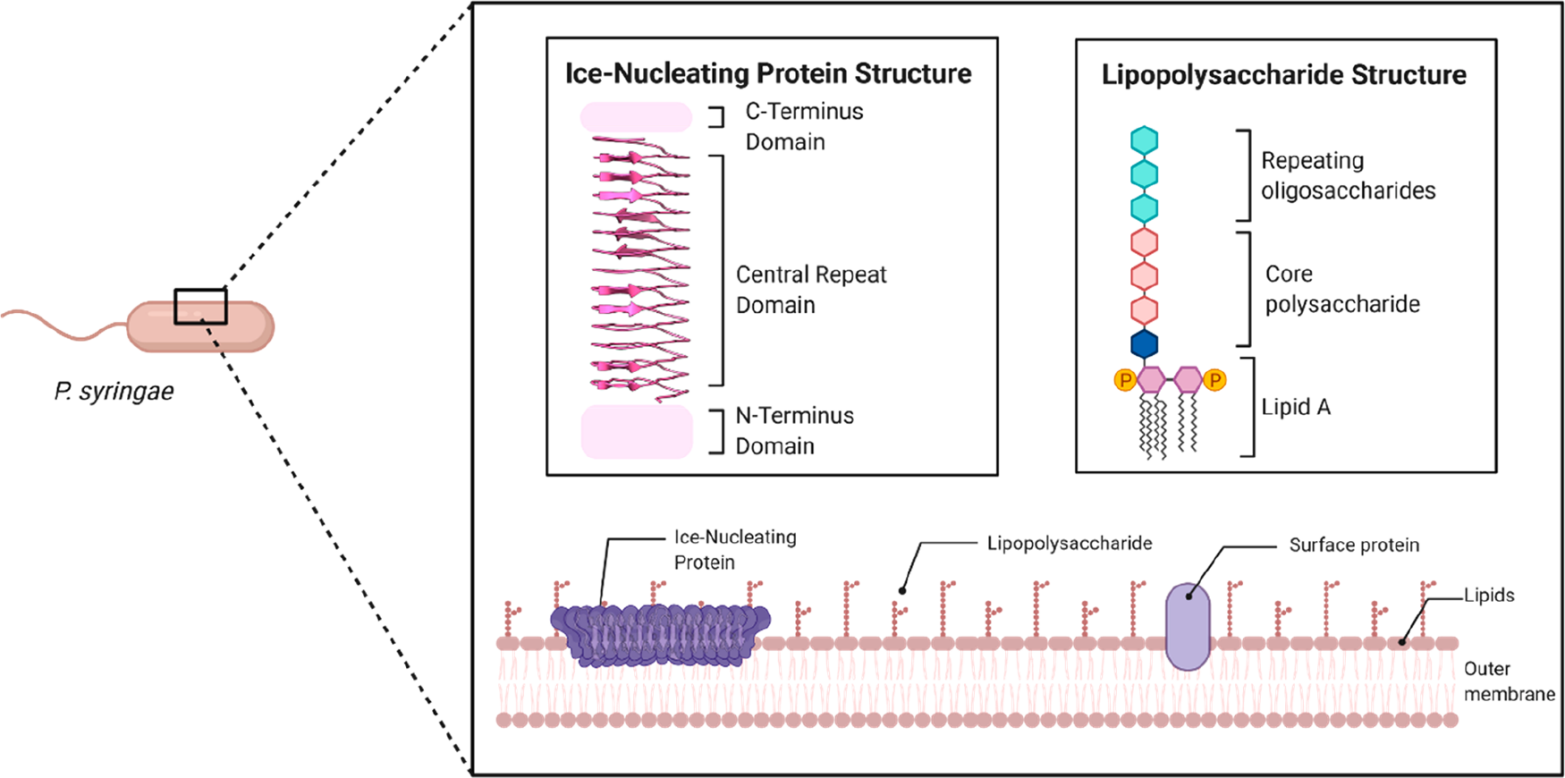
Schematic representation of the Gram-negative, rod-shaped bacterium *P. syringae*, the modeled structures of an ice-nucleating protein, and a lipopolysaccharide, as well as their location in the outer bacterial cell membrane.

Figure 2a shows the results of ice nucleation measurements of the bacterial INs Snomax in water. Snomax consists of inactivated cells of *P. syringae* and is widely used as a model for bacterial ice nucleation studies.^22^ The initial *P. syringae* solutions had a concentration of 0.1 mg/mL and were then serially diluted 10-fold, resulting in concentrations from 1 ng/mL to 0.1 mg/mL. The cumulative ice nucleator number concentration (*N*_m_) was calculated using Vali’s formula and represents the number of ice nucleators per unit weight that are active above a certain temperature.^23^ For the bacterial IN solution in water, the spectrum shows two strong increases in *N*_m_*(T)* around ∼−2.9 and ∼−7.5 °C with plateaus between ∼−4.5 and ∼−7 °C and below ∼−9.5 °C. The two rises in the spectrum reveal the presence of two classes of IN with different activation temperatures. We attribute the observed rises at ∼−2.9 and ∼−7.5 °C to class A and C IN, respectively. Class A IN supposedly consist of large INP assemblies and the less active class C IN consist of smaller INP assemblies. We purified the INPs of *P. syringae* using Folch extraction (FE) and ice affinity purification (IAP). FE is based on the partitioning of lipids in a biphasic mixture of chloroform and methanol and causes a separation of lipid and protein components.^24^ IAP uses the ability of INPs to bind to ice, and the purification process involves the incorporation of INPs into the growing ice phase and the exclusion of impurities.^20,25^ We will refer to the purified samples as “lipid” (FE extraction) and “purified INP” (FE extraction and subsequent IAP) fractions. Figure 2 shows that the freezing spectra of both the lipid and purified INP fractions of *P. syringae* look different than the spectrum of *P. syringae* in water. For both fractions, we observe that the class A related increase at ∼−2.9 °C is absent and that the total number of INs is reduced. For the purified INPs, we observe an increase at ∼−7 °C, and for the lipid fraction, an increase at ∼−8 °C. Apparently, the removal of the lipids keeps the class C INs largely intact, but it prevents the formation of the highly efficient class A INs. Interestingly, the lipid fraction retained significant activity. Ice nucleation activity of lipids alone can be excluded, as demonstrated by Figure 3, which shows a lack of IN activity for different lipids and lipopolysaccharides (LPS). We thus explain this observation with remaining INPs in the lipid fraction. Figure 2 also shows the results of combining the purified INPs with the lipid fraction. We find that the freezing spectra of the combined INP+lipid fractions show an increase at ∼−7 °C corresponding to class C INs and no increase at ∼−2.9 °C. *P. syringae* samples that underwent delipidation treatment but were not separated also only showed an increase at ∼−7 °C corresponding to class C INs and no increase at ∼−2.9 °C (Figure S1).

**Figure 2:**
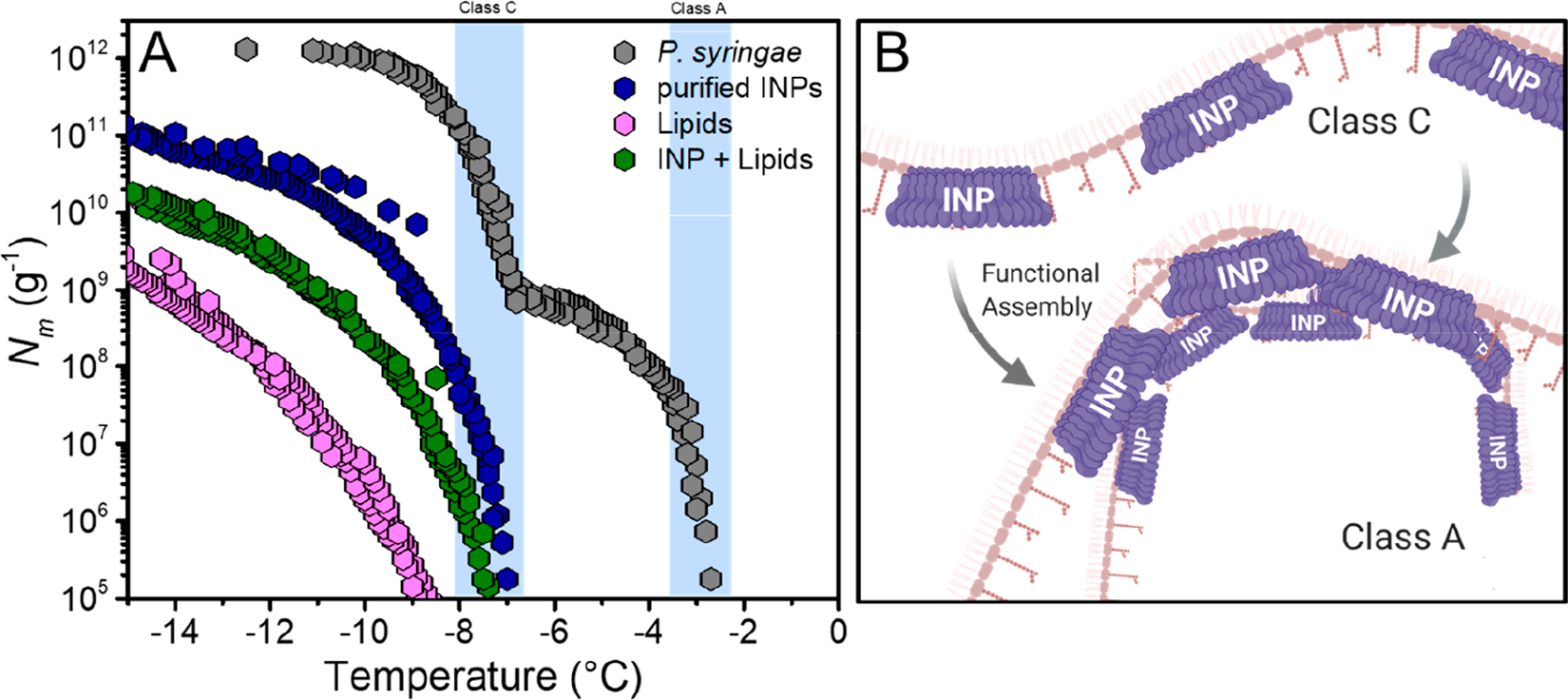
Freezing experiments with aqueous solutions of *P. syringae,* purified INPs, extracted lipids, and a combination of purified INPs and lipids in water. (A) Cumulative number of ice nucleators per unit mass of sample (*N*_m_) plotted against temperature. The temperature ranges for class A and C bacterial ice nucleators in water are shaded in blue. (B) Hypothetical representation of class A and C ice nucleators in a membrane. Class C refers to small INP assemblies and class A to larger, highly efficient INP assemblies.

**Figure 3:**
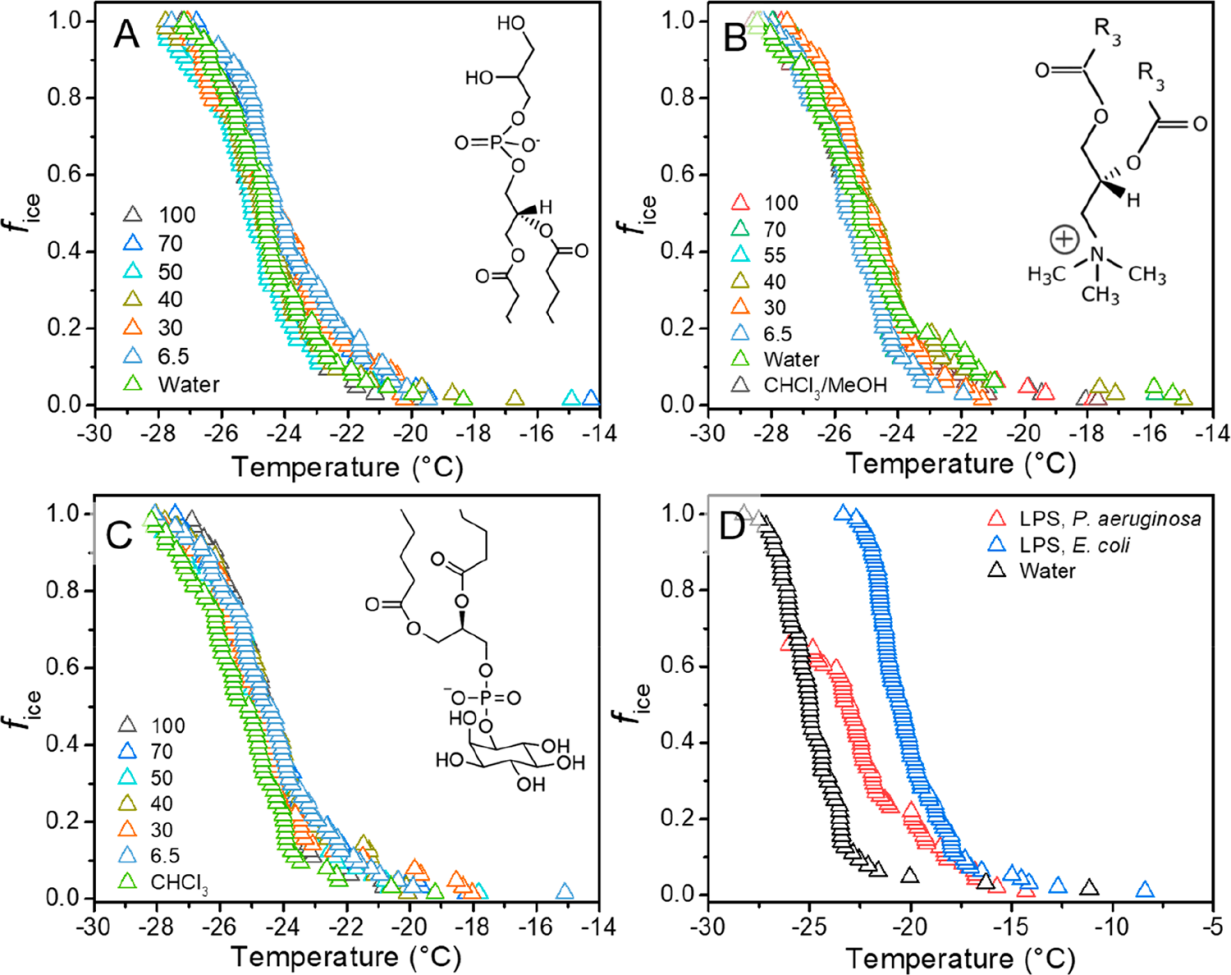
Droplet freezing experiments of phospholipids and LPS. Parts A–C show the fraction of frozen 1 μL droplets plotted against temperature, for the lipids DPPE, DPPG, and PI, respectively. Lipids were dissolved in 9:1 chloroform:methanol and measurements were performed with surface coverages ranging from 6.5 to 100 Å^2^/molecule on water. (D) Freezing curves of aqueous LPS solutions (5 mg/mL) extracted from ice-nucleation active and nonactive bacteria. Insets show the chemical structure of the lipid headgroups.

Clearly, the loss of activity of class A INs upon delipidation is irreversible, and reintroducing a lipid matrix is insufficient to restore activity of class A INs.

Figure 3 shows the results of statistical freezing curves of the lipids DPPE, DPTAP, PI, DPPG (Figure S2), and LPS. The activities of the lipids were determined in aqueous solutions with different surface coverages (for details, see methods). We find that all of the lipids show negligible ice nucleation activity. The observed *T*_50_ values of ∼−25 °C are similar to those of pure water in our setup. *T*_50_ values are defined as the temperatures at which 50% of the droplets are frozen. These observations are in line with previous findings that long-chain fatty acids and surfactants are poor INs.^26,27^

LPS is another major component of the outer layer of the cell membrane of Gram-negative bacteria. LPS molecules are often associated with membrane proteins and are in direct contact with the environment. Figure 3d shows freezing spectra of LPS extracts from the ice nucleation-active bacteria *Pseudomonas aeruginosa*(28) and the nonactive bacteria *Escherichia coli*. At maximal LPS concentrations of 5 mg/mL, the ice nucleation activity is weak, with freezing occurring at *T*_50_ = ∼−22.5 °C for *P. aeruginosa* and ∼−20 °C for *E. coli*. LPS has previously been reported to have moderate ice-nucleating abilities, which agrees with our findings.^29,30^ We conclude that LPS might play minor roles in ice nucleation, but since LPS derived from the nonactive bacteria showed higher activity, this role is likely not crucial for the class A IN that enables maximum freezing efficiency.

Having established that lipids and LPS are not active components of the ice nucleation site, we hypothesized whether the role of the membrane might be to serve as a functional assembly matrix for the highly efficient class A IN. Figure 4 shows the results of ice nucleation measurements of the bacterial IN in water, deuterated water (D_2_O), and in mixtures of the two. For *P. syringae* in pure D_2_O, the freezing spectrum looks similar to that of *P. syringae* in water, with a ∼4 °C shift of the INP-mediated freezing curve to warmer temperatures. The observed shift is consistent with the expected shift of ∼3.82 °C based on the higher melting point of D_2_O. Turner et al. previously described a third intermediate class B INs, active at around ∼−5 °C, and that examining the effects of substituting D_2_O for H_2_O allows for differentiation of the different classes on the basis of their isotope-induced shifts in nucleation threshold.^13^ As apparent from Figure 4, the freezing spectra did not show an additional increase assignable to a third class of INs. However, we did observe some differences in the freezing curves of *P. syringae* in H_2_O and D_2_O. We explain the observed differences with higher rigidities of INPs in D_2_O and fewer structural fluctuations of the INP-assemblies due to the stronger intramolecular D-bonds.^31,32^ In fact, at a macroscopic level, there is some evidence suggesting that D_2_O is a worse solvent than water and that proteins tend to reduce the surface area in contact with D_2_O by forming larger aggregates.^33,34^ Thus, especially small INP aggregates, present at lower concentrations, tend to increase their size and thereby the ice nucleation efficiency as shown in Figure 4B

**Figure 4:**
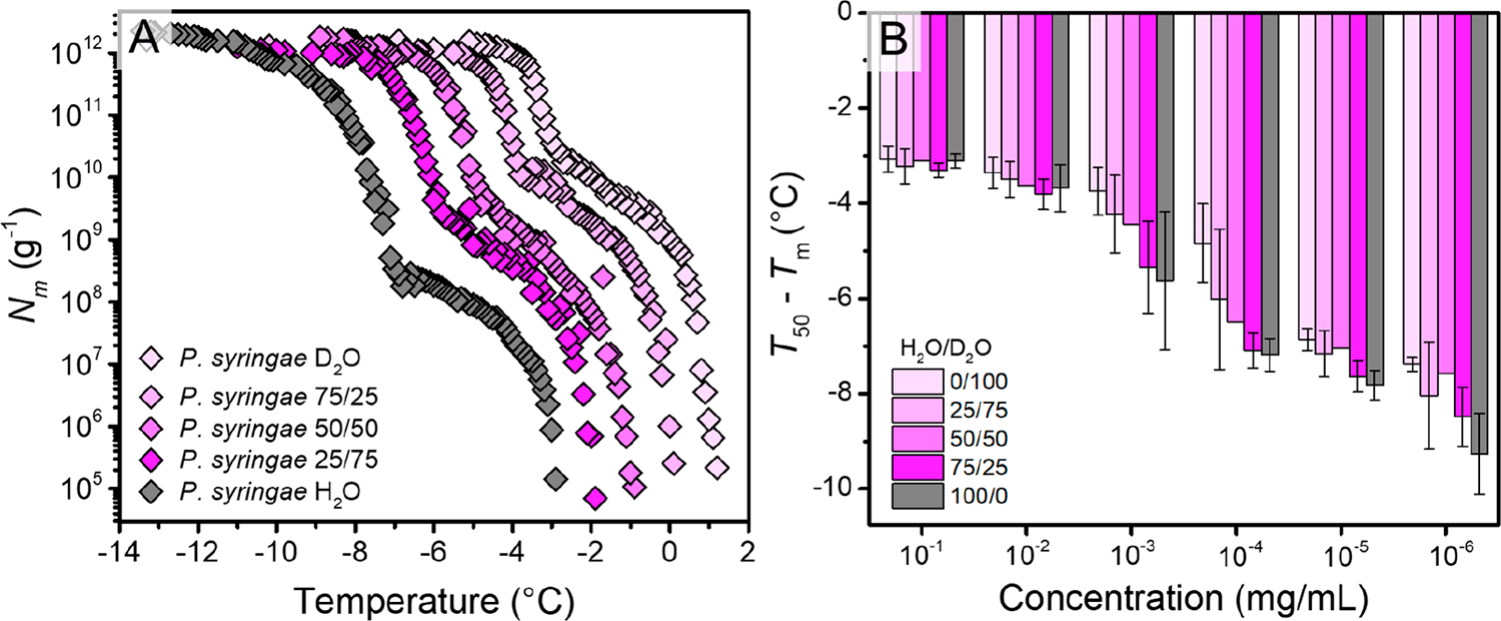
Freezing experiments of aqueous solutions of *P. syringae* in water, deuterated water, and mixtures of the two. (A) Cumulative number of ice nucleators (*N*_m_) per unit mass of sample vs temperature. (B) *T*_50_ values of *P*. *syringae* in water and D_2_O mixtures, corrected for their respective melting points. *T*_50_ values are defined as the temperatures at which 50% of the droplets are frozen.

INPs are localized in the outer bacterial membrane, suggesting that lipids might be required for ice nucleation activity as part of the active nucleation site or by enabling precise INP assembly. The fact that we did not observe ice nucleation activity of lipids and LPS components of the membrane implies that they are not part of the nucleation site. Interestingly, PI was previously reported to be an important component in ice nucleation sites of bacteria and insects.^15,16,35^ In these studies, the presence of borate compounds was further shown to dramatically reduce the ice nucleation activity of *P. syringae,* which was explained by the complexation of the hydroxyl groups of the inositol.^6,36^ We find that PI shows no ice nucleation activity, and the presence of borate compounds also did not reduce the ice nucleation activity of *P. syringae* (Figure S3). Our findings further support the interpreation that class C and class A INs from *P. syringae* do not differ in the structure of their INP building blocks but solely in their assembly size and supramolecular ordering. After delipidation experiments, the purified INPs displayed ice nucleation activity that could be assigned to class C INs, while class A activity was completely removed. We conclude that class C IN only consists of small INP assemblies that do not require lipids for functionality, whereas class A aggregates require an intact membrane environment for functional alignment and aggregation. Using different H_2_O/D_2_O ratios, we were able to show that the extent of the assembly of INPs and the corresponding amount of class A and class C INs can be altered depending on the solvent mixture. Our results unambiguously prove that maximum ice nucleation activity observed in bacteria results from the association of INPs within the membrane. Hence, studies that take only the bacterial INPs into consideration have to be taken with a caveat, as they do not resemble the bacterial system and their highly efficient INs. Our data is consistent with a mechanism, in which bacteria have to exert precise control over (1) the distance between the INP monomers at the sub-Ångstrom level, and (2) the size of the protein assemblies to achieve high ice nucleation activities.^10^ This mechanism explains the high sensitivity of class A INs to temperature as well as chemicals^7,11,17,19^ that modify the properties of the cell membrane or the aggregation behavior of proteins. Given that highly active class A INs have also been observed in mutated *E. coli* cells^12^ suggests that INP assembly is robust toward variations in exact membrane composition.

## Supporting Information Available

Experimental methods; Figure S1, effect of Folch extraction on bacterial INs; Figure S2, droplet freezing experiments of the phospholipid DPPG; Figure S3, effect of borate compounds (0.3 M) on bacterial INs (PDF)
