# Supplementary material for: Membranes Are Decisive for Maximum Freezing Efficiency of Bacterial Ice Nucleators
Source: J Phys Chem Lett. 2021 Nov 1;12(44):10783–7. doi: 10.1021/acs.jpclett.1c03118 (PMC8591660; doi:10.1021/acs.jpclett.1c03118)
Supplement: Supplementary file 1 — jz1c03118_si_001.pdf [file jz1c03118_si_001.pdf]

Supporting Information

Title: Membranes Are Decisive for Maximum Freezing Efficiency of Bacterial Ice Nucleators

Authors: R. Schwidetzky[1], P. Sudera[1], A. T. Backes[2], U. Pöschl[2], M. Bonn[1], J. Fröhlich-Nowoisky[2], and K. Meister[1,3]

Affiliations:

[1]Max Planck Institute for Polymer Research, 55128 Mainz, Germany

[2]Max Planck Institute for Chemistry, 55128 Mainz, Germany

[3]University of Alaska Southeast, 99801 Juneau, AK, United States

Methods:

Materials. Pure water was obtained from Millipore Milli-Q® Integral 3 water purification system (Merck Chemicals GmbH, Darmstadt, Germany), autoclaved at 121 °C for 15 min, and filtered through a 0.1 µm bottle top filtration unit (VWR International GmbH, Darmstadt, Germany). Deuterated water, lipids, and lipopolysaccharide extracts were purchased from Sigma Aldrich. Snomax® was purchased from SMI Snow Makers AG (Thun, Switzerland) and consists of a preparation of inactivated bacteria cells of *P. syringae*.

Folch Extraction. Folch extraction was performed by a protocol adapted from Wessel *et al*[37]. In short, *P. syringae* was dissolved in water at a concentration of 10 mg/mL. 5 mL of the solution was pipetted in a 50 mL falcon tube, 20 mL methanol was added, and the mixture was vortexed thoroughly. Then, 10 mL chloroform was added, and the solution was vortexed again. After the addition of 15 mL water, the mixture was generously vortexed and centrifuged at 13,000 g for 1 min. The resulting sample contained a large aqueous layer on top, a circular flake of protein in the interphase, and a smaller chloroform layer at the bottom. The upper layer was carefully removed, 15 mL methanol was added, vortexed, and centrifuged at 13,000 g for 2 min. All samples were dried under vacuum.

Ice Affinity Purification. Rotary ice-shell purification was used to purify the ice-nucleating biomolecules of Snomax. Details of the purification method have been described elsewhere[20, 25]. In short, ~20–30 mL of water was used in a 500 mL flask to form an ice-shell using a dry ice-ethanol bath for 30–60 s. The flask was then rotated in a temperature-controlled ethylene glycol bath, and the temperature of the bath was set to −2 °C. 50 mL precooled bacterial IN solution was added, and the flask rotated continuously in the bath until 30% of the solution was frozen. The obtained ice was melted and freeze-dried to obtain a mixture of present ice-binding proteins from *P. syringae*.

TINA Experiments. Ice nucleation experiments were performed using the high-throughput Twin-plate Ice Nucleation Assay (TINA), which has been described in detail elsewhere[21]. In a typical experiment, the investigated IN sample was serially diluted 10-fold by a liquid handling station (epMotion ep5073, Eppendorf, Hamburg, Germany). 96 droplets (3 µL) per dilution were placed on two 384-well plates and tested with a continuous cooling-rate of 1 °C/min from 0 °C to −30 °C in H_2O and 5 °C to -25 °C in D_2O with a temperature uncertainty of ±0.2 °C. The droplet-freezing was determined by two infrared cameras (Seek Therman Compact XR, Seek Thermal Inc., Santa Barbara, CA, USA). The obtained fraction of frozen droplets was used to calculate the cumulative number of ice nucleators using the Vali formula[23]. Experiments were performed multiple times with independent samples. The exact number of independent measurements were Figure 2 (INP extract: six experiments, lipid extract: three experiments, INP + lipid: three experiments); Figure 3 (all experiments were performed in duplicates); Figure 4 (100% D_2O: three experiments, 100%: H_2O three experiments, mixtures: two experiments). Background freezing of pure water in our system occurred at ~−25 °C. For the TINA lipid experiments, a 0.18 x 10^{-3} M stock solution was prepared for all lipids by dissolving the respective lipid in a 9:1 chloroform/methanol solution. Considering the vial diameter of the 384-well plate to be 3 mm wide in the center, and using the surface area of a circle, molar concentrations for different surface coverages of the lipids were calculated, considering a 30 µL drop volume. Surface areas of interest ranged from 30 Å^2/molecule (0.039 x 10^{-3} M) to 100 Å^2/molecule (0.0117 x 10^{-3} M), and larger values correspond to lower concentrations and a waiting time of 15 min after addition of samples was maintained to allow for solvent evaporation. Lipid and LPS extract experiments were performed twice with independent samples.

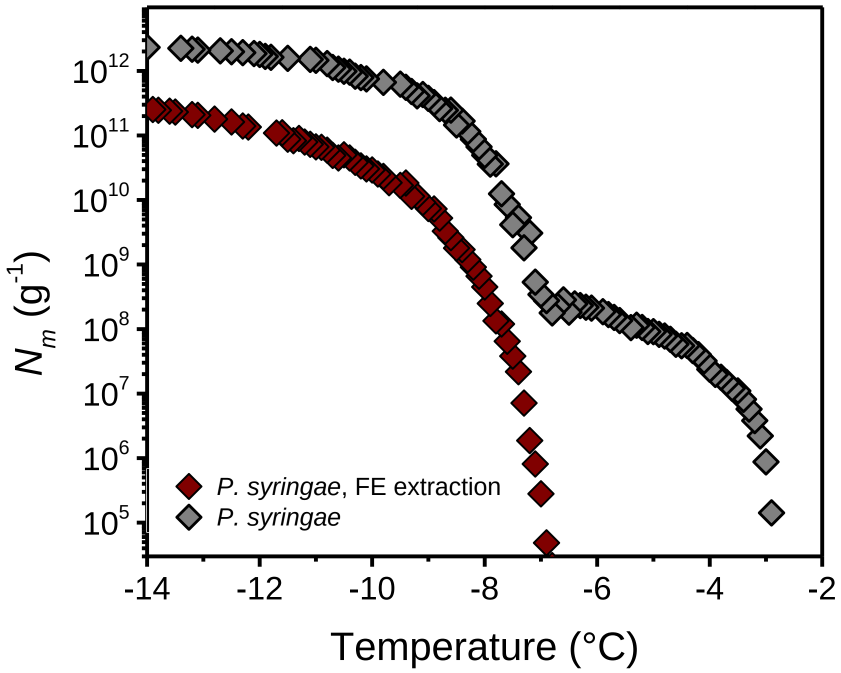

Figure S1 Effect of performing a Folch extraction (FE) on the ice nucleation activity of *P. syringae*. The lipid and proteins fraction were not separated and the organic solvents were evaporated. Shown are the cumulative number of ice nucleators per unit mass of sample (N_m) plotted against temperature.

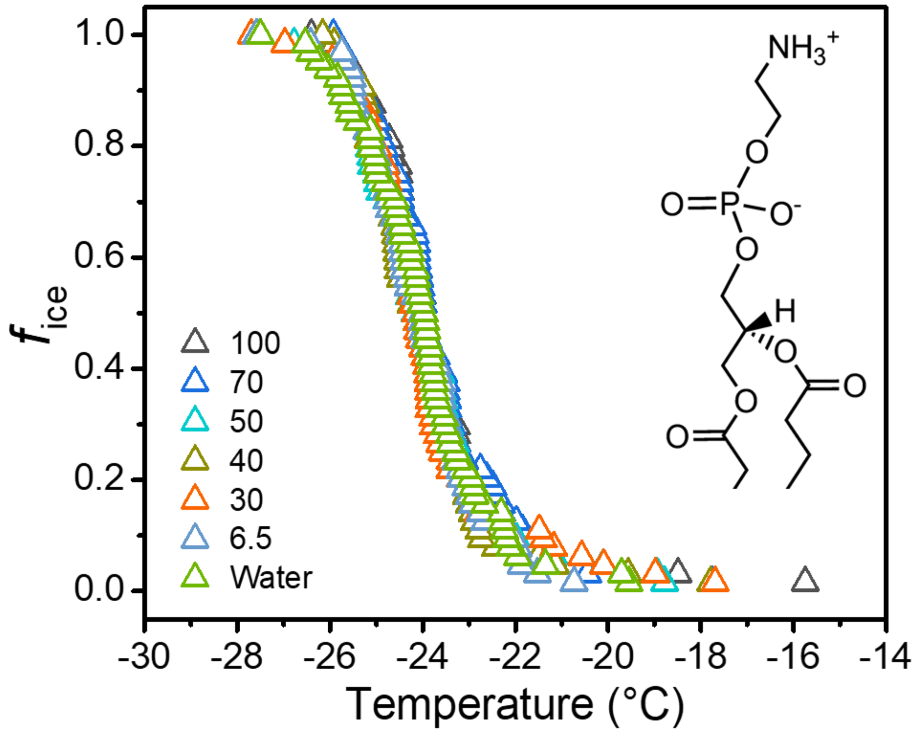

Figure S2 Droplet freezing experiments of the phospholipid DPPG. Shown are the fraction of frozen 1 μL droplets plotted against temperature. DPPG was dissolved in 9:1 chloroform:methanol and measurements were performed with surface coverages ranging from 6.5 to 100 Å^2/molecule on water.

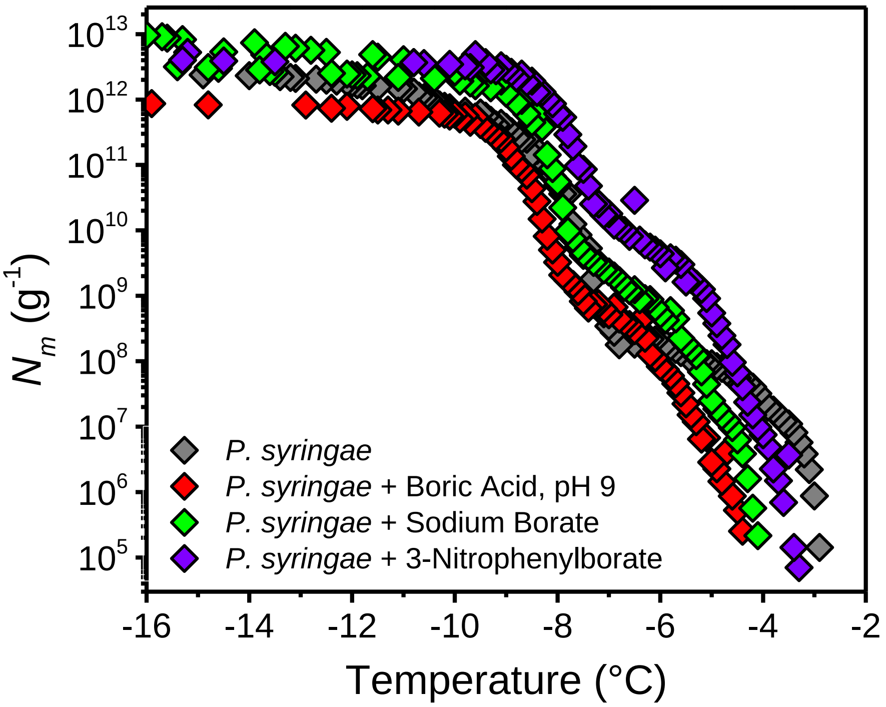

Figure S3 Effect of different borate compounds (0.3 M) on the ice nucleation activity of *P. syringae*. Shown are the cumulative number of ice nucleators per unit mass of sample (N_m) plotted against temperature.
